# Supplementary material for: Digital transformation and liquidity creation in commercial banks: Evidence from the Chinese banking industry
Source: PLoS One. 2025 Feb 13;20(2):e0318785. doi: 10.1371/journal.pone.0318785 (PMC11825035; doi:10.1371/journal.pone.0318785)
Supplement: Attachment — Submitted filename: review.docx [file pone.0318785.s001.docx]

**Language and Style:**

Ensure the paper is well-written, concise, and free from grammatical errors.

Maintain a consistent writing style and tone throughout the paper.

**Theoretical Contributions:**

Explicitly outline the theoretical contributions your study makes to the field. How does your research advance our understanding of the relationship between fiscal competition and innovation investment?

**Introduction Clarity:**

Provide a comprehensive introduction that clearly outlines the research problem, the significance of the study, and the objectives. Clearly state the rationale for examining the relationship between fiscal expenditure competition and innovation investment.

**Theoretical Contributions:**

Explicitly outline the theoretical contributions your study makes to the field. How does your research advance our understanding of the relationship between fiscal competition and innovation investment?
