# Supplementary material for: Digital transformation and liquidity creation in commercial banks: Evidence from the Chinese banking industry
Source: PLoS One. 2025 Feb 13;20(2):e0318785. doi: 10.1371/journal.pone.0318785 (PMC11825035; doi:10.1371/journal.pone.0318785)
Supplement: Attachment — Submitted filename: Response to Reviewer3.docx [file pone.0318785.s002.docx]

**Response to Reviewer 3 Comments**

Dear Editors and Reviewers:

Thank you very much for taking your time to review and comments concerning our manuscript entitled “Digital transformation and liquidity creation in commercial banks: evidence from the Chinese banking industry”. Those comments are all valuable and very helpful for revising and improving our paper. We have studied comments carefully and have made correction and point-by-point response which we hope meet with approval. Our responses are detailed below and highlighted in red. The main changes in the paper and the responds to the reviewer #3 are as follows:

Q1: The introduction should mention the motivations to conduct this study in China, as prior studies such as (Jiang Hai et al., 2023), (Guo Ye et al., 2022), (Li Yifei et al., 2022) address the digital transformation index in China. What are the unique institutional characteristics about Liquidity creation and digital transformation in China?

Response: We thank reviewer for reminding us this important point. We add the liquidity problem faced by China's financial market, which is very different from that of other countries, to introduce the necessity of the integration of digital technology and traditional finance and the main issues studied in this paper. The details are as follows:

“*However, for a long time, China's real economy has been faced with financing difficulties and high financing costs, while the problem of idling arbitrage in the banking system still exists. In response to this problem, in recent years, the regulatory authorities have issued a few policies, such as making banks that meet the assessment requirements of inclusive finance enjoy a certain preferential deposit reserve ratio, reducing excess deposit reserves to release signals guiding credit expansion, etc. These measures have alleviated the shortage of funds in the real economy in the short term, but this problem has always been difficult to be substantively resolved.” (LINE 64-71)*

Q2: The contribution needs citations and explain how this study extends prior studies in data, method and idea.

Response: Thank you very much for this comment. We have revised the innovation part of this paper as follows.

“*Firstly, existing literature has begun to pay attention to the impact of digital transformation of banks on banking operations and the real economy, but the investigation on this issue focuses on the asset side of banks. The index of bank liquidity creation is a comprehensive measure of the smooth conduction of liquidity through the banking system by the asset side and the liability side of the bank. Therefore, from the perspective of liquidity creation, it is more comprehensive to examine the issue that the digital transformation of banks can help improve the efficiency of banks to serve the real economy. Secondly, the existing literature on the connection between digital transformation and mobility creation needs to be supplemented, both theoretically and empirically. This paper focuses more comprehensively on the potential for banks' digital transformation to reduce or increase liquidity creation through multiple transmission channels. At the theoretical level, the paper makes a detailed analysis of how the digital transformation of banks affects liquidity creation, and at the same time, it makes a more detailed test of the internal relationship between the two through empirical research. Thirdly, considering that there are considerable differences in the development of different types of banks and different regional banks in China's banking system, based on the development status of Chinese banks, this paper reveals the impact of digital transformation on liquidity when banks face different external environments and different internal conditions. It provides reliable empirical evidence for banks to formulate digital transformation strategies in accordance with local conditions in China.” (LINE 114-132)*

Q3: The literature should incorporate the theories that explain the nexus between digital transformation and liquidity creation. For example: Vuong, G. T. H., Phan, P. T. T., Nguyen, C. X., Nguyen, D. M., & Duong, K. D. (2023). Liquidity creation and bank risk-taking: Evidence from a transition market. Heliyon, 9(9).

Response: We sincerely appreciate the valuable comment. In the second chapter, literature review and research hypothesis are reviewed, and the relationship between digital transformation and bank liquidity creation is directly emphasized. Specifically as follows：

*“In recent years, the continuous integration of digital technology represented by big data, cloud computing and artificial intelligence with the financial industry has reshaped and promoted the innovation of financial products and service models, resulting in major changes in the business behavior and structure of banks (Liu, 2016; Guo and Zhu, 2021). These changes have had a profound impact on the level of liquidity creation within banks and their impact on the real economy (Song et al., 2023).” (LINE 164-169)*

Q4: The variable definitions should include variable definition tables to summary how authors calculate the variables.

Response: Thanks for this comment. We added the measurement of variables in the descriptive statistics table to make the definition of variables more intuitive. At the same time, the profitability statistics are supplemented.

| Variable | symbol | Method of measurement | Mean value | Standard deviation | Minimum value | median | Maximum value |
| --- | --- | --- | --- | --- | --- | --- | --- |
| liquidity creation | Lc | Constructed according to the three-step cat-fat classification method proposed by Berger and Bouwman (2009) | 54.345 | 17.421 | 24.078 | 51.436 | 192.160 |
| Digitization of Banking Services | Tdi | Peking University Digital Transformation Index of Chinese commercial banks | 52.137 | 38.468 | 0 | 52 | 200 |
| Provision for loan losses | Llp | Loan loss reserve/total loans | 0.033 | 0.937 | 0 | 0.051 | 0.152 |
| Risk tolerance | Risk | Risk-weighted assets/total assets | 0.587 | 5.342 | 0.223 | 0.649 | 0.915 |
| Financial disintermediation | Findis | (regional corporate bonds + non-financial corporate stocks)/social financing scale | 0.156 | 3,552 | 0.375 | 0.147 | 0.08 |
| Industrial structure | Ind | Gross secondary industry product/Gross regional product | 46.743 | 9.451 | 32.762 | 48.702 | 81.328 |
| Business cycle | M2 | M2 growth rate | 11.460 | 2.346 | 6.990 | 11.010 | 18.950 |
| Level of regional economic development | Gdpg | Regional GDP growth rate | 10.768 | 0.654 | 9.299 | 10.739 | 12.123 |
| Non-performing loan ratio | Npl | Non-performing loans/total loans | 1.627 | 0.764 | 0.049 | 1.214 | 4.376 |
| Capital adequacy ratio | Tcr | Total capital/risk-weighted assets | 13.449 | 3.135 | 0.018 | 12.771 | 57.610 |
| Asset size | Size | Ln (Total assets) | 25.674 | 1.529 | 22.391 | 25.395 | 31.138 |
| Percentage of non-interest income | Nir | Non-interest income/operating income | 34.216 | 10.159 | 0 | 33.315 | 206.077 |
| Profitability | *Roe* | Net profit/net assets | 15.172 | 6.383 | 0.813 | 13.624 | 32.469 |

Q5: In the baseline models, the authors employ the FEM method, how could the results are free from autocorrelation and heteroskedasticity issues?

Response: Thanks for this comment. To control heteroscedasticity and autocorrelation problems, we Cluster all the standard errors of regression coefficients at the bank level. It is explained in P10.

*“To control for potential heteroscedasticity and serial correlation, all regression coefficients are clustered and have robust standard errors.” (LINE 460-461)*

Q6: Honestly, I loose my focus as the study incorporate so many objectives. It would be better for readers if the authors narrow down the focus and consolidate the content of the study.

Response: Thanks for this great point. We have deleted some unnecessary phrases and other descriptions in the paper. For example, first, we simplify the language in the introduction and find the entry point directly through existing problems; (P2-5)

Second, in the empirical results part, I asked to test the analysis part of each table and analyze the empirical results combined with the economic reality; (P21-36)

Third, to make the research question clearer, we re-adjusted the mechanism of action diagram.

Q7: The findings needs in-depth and economic discussion, rather than describe the tables.

Response: Indeed, the discussion on the limitations of the study was ignored. In the table test analysis part of the empirical results, we analyze the empirical results combined with the economic reality to help understand the research problem in this paper.

*“Column (1) only considers the digital transformation of banks as a regression variable, and column (2) adds control variables. The results show that there is a significant positive correlation between digital transformation and liquidity creation, and indicates that the increase of bank liquidity creation is related to the process of digital transformation of banks. Our results are consistent with existing research demonstrating that the integration of digital technology and finance can help banks expand long-tail customers (Zhang and Naceur, 2019), increase loan volume (Sheng, 2021) and increase bank credit supply, thereby increasing bank liquidity creation. So, hypothesis 1 is tested.” (LINE 486-493) (LINE 64-71)*

*“Due to the application of digital technology, commercial banks have improved the ability to monitor loan credit risks, improved the forward-looking provision for credit losses, and avoided excessive provision for loan losses to cushion risks.” (LINE 546-548)*

*“This is due to the application of advanced technology in bank business activities, which effectively improves the information transparency of lending enterprises and reduces banks' concerns about unexpected risks. Therefore, under the premise of controllable risks, banks tend to obtain more interest spread returns by taking certain risks voluntarily, which is ultimately reflected in the increase of risk tolerance.” (LINE 565-569)*

*“Because banks use digital technology to lower the threshold of financial services, expand loan services for small and micro enterprises, agriculture and vulnerable groups, ease the financing constraints of these groups, and promote the development of the real economy.” (LINE 586-589)*

*“The possible reason is that in regions with perfect digital infrastructure, banks can take advantage of superior conditions to promote their own digital transformation, and advance the use of advanced technology to innovate financial products to enhance their competitiveness, thus promoting the creation of bank liquidity.” (LINE 718-721)*

*“This suggests that the development of digital finance will weaken the positive relationship between the digital transformation of banks and the creation of liquidity, which may be because the development of digital finance, while improving the efficiency of financial services and stimulating the digital transformation of commercial banks, will give birth to more financial service platforms to seize the credit market, form a new competitive pattern, and reduce the credit market share of commercial banks.” (LINE 739-744)*

*“This may be because banks with high resource endowments typically have higher levels of capitalization, can facilitate the use of fintech for business transformation and product innovation, and have strong borrowing capacity, thus creating more liquidity (Fang et al., 2023).” (LINE 769-772)*

*“This may be because the credit structure of large state-owned banks and joint-stock banks shows the characteristics of concentrated customers, high proportion of large loans, and systematic business development. With the support of digital technology, large banks have more competitive advantages in marketing, risk assessment and other aspects. Reducing the operating cost of bank credit business through economies of scale is helpful to improve the willingness of banks to invest liquidity in the medium and long term, thus improving the level of liquidity creation (Xiang and Gao, 2023).” (LINE 788-794)*

8. There are many interesting results and the implications should closely linked with the main findings.

Response: Thanks for this great point. In the conclusion part of the sixth chapter, we reorganize and put forward corresponding policy suggestions according to the research conclusions of this paper

*“First, the shortage of bank liquidity has hindered the transmission of monetary policy to a certain extent, and the digital transformation of banks can help improve the level of bank liquidity. Therefore, regulatory authorities should encourage commercial banks to promote the process of digital transformation, and promote the deep integration of fintech, commercial banks and the real economy through policy support. (LINE 814-818)*

*Secondly, it is considered that the digital transformation of banks can alleviate the liquidity shortage by optimizing loan loss provisions, improving risk tolerance and restraining financial disintermediation. Commercial banks should make use of digital technology to reconstruct financial logic and transform service mode to improve their risk resistance ability. At the same time, banks need to personalize customer service and develop new customers through digital means, to improve the internal power of banks' digital transformation to serve the real economy. (LINE 826-843)*

*Finally, due to the heterogeneity of the effects of banking digitalization on liquidity creation, regulatory authorities should respond with differentiated measures.” (LINE 844-845)*

This letter is our point-by-point response to the comments raised by the reviews. The comments are produced and our responses are given directly afterward in a different color from the authors in the revised copy.

We would also like to thank you for allowing us to resubmit a revised copy of the manuscript.

We hope that the revised manuscript may address the concerns raised by the reviewers and will have the opportunity to be published in PLOS ONE.
