# Supplementary material for: Digital transformation and liquidity creation in commercial banks: Evidence from the Chinese banking industry
Source: PLoS One. 2025 Feb 13;20(2):e0318785. doi: 10.1371/journal.pone.0318785 (PMC11825035; doi:10.1371/journal.pone.0318785)
Supplement: Attachment — Submitted filename: Response to Reviewer.docx [file pone.0318785.s003.docx]

**Response to Reviewer 3 Comments**

Dear Editors and Reviewers:

Thank you very much for taking your time to review and comments concerning our manuscript entitled “Digital transformation and liquidity creation in commercial banks: evidence from the Chinese banking industry”. Those comments are all valuable and very helpful for revising and improving our paper. We have studied comments carefully and have made correction and point-by-point response which we hope meet with approval. Our responses are detailed below and highlighted in red. The main changes in the paper and the responds to the reviewer #3 and editors are as follows:

Q1:  Reviewer and editors specifically suggesting that the paper be proofread by a professional language editor before submission.

Response: We thank reviewer for reminding us this important point. We made some modifications to the expressions in the paper. We also sought the help of a professional language editor to conduct a further review and revision of the paper's language expression.

Q2: The mediation analysis presented does not include a formal test for mediation. It would be beneficial to revisit the methodology section to strengthen the discussion on mediation and incorporate a formal test. The analysis should be adjusted accordingly.

Response: Thank you very much for this comment. We re-examined and corrected the existing measurement models, and re-evaluated the mediation methods mentioned in the paper. At the same time, we considered and analyzed all possible scenarios that could arise in the mediation model.

“*Models (2), (3) and (4) collectively constitute the equations for the intermediation effect test. Generally, three steps are employed to test the intermediate variables. Taking the mediating effect of testing risk tolerance as an example, the first step is to conducting an empirical estimation of model (2) to evaluate the overall impact of banks' digital transformation on liquidity creation without considering risk tolerance. If* $\alpha_{1}$ *is significant, it suggests the existence of the overall effect of digital transformation on liquidity creation exists. In the second step, model (3) is estimated empirically. If the coefficient* $\beta_{1}$ *is significantly positive, it indicates that the digital transformation will improve the bank's risk tolerance; if the coefficient* $\beta_{1}$ *is significantly negative, it indicates that the digital transformation diminishes the bank's risk tolerance, and then the following analysis will be carried out; otherwise, the test is terminated. The third step is to conducting an empirical estimate of model (4) to examine whether the impact of bank digital transformation on liquidity creation changes with the introduction of bank risk tolerance factor. If* $\alpha_{1}$ *is not significant, it indicates the absence of an intermediary effect. If* $\alpha_{1}$ *is significant, and* $\beta_{1}$ *and* $\gamma_{2}$*are also significant, this indicates that there is an intermediate effect. If* $\alpha_{1}$ *is significant while* $\gamma_{1}$ *is not, it indicates a complete mediation effect. If* $\gamma_{1}$ *and* $\gamma_{2}$ *coefficients are both significant, it indicates that there is a partial intermediary effect. If* $\beta_{1}$ *and* $\gamma_{2}$ *are not completely significant, then the Z-statistic constructed by Sobel method can be further tested. If the null hypothesis is rejected: The regression of model (3) is carried out based on model (2). If the coefficient β_1 is significantly positive, it indicates that digital transformation will improve the risk tolerance of banks, and then the following analysis will be carried out; otherwise, the test will be terminated. Subsequently, model (4) is estimated. If the coefficients* $\gamma_{1}$ *and* $\gamma_{2}$ *are both significantly positive, it indicates that the digital transformation plays a partial mediating role in influencing the risk tolerance of banks in the process of liquidity creation. If* $\gamma_{1}$ *is not significant and the* $\gamma_{2}$ *coefficient is significantly positive, it indicates that the risk tolerance plays a complete mediator. If the coefficients* $\gamma_{1}$ *and* $\gamma_{2}$ *are not significant, then there is no intermediary effect. The intermediation effect test of loan loss provision and financial disintermediation follows the same logic as described above.” (LINE 725-757)*

*“The intermediary effect test confirms that the digital transformation of banks can reduce provisions for loan losses, ultimately enhancing liquidity creation levels. This indicates that provisions for loan losses serve as an intermediary in the process of improving liquidity generation through banks' digital transformation. Thus, Hypothesis 2 is supported.” (LINE 918-944)*

*“The intermediary effect test confirms that the digital transformation of banks enhances liquidity creation by improving their risk tolerance, indicating that risk tolerance functions as an intermediary in the process through which digital transformation elevates liquidity levels. This finding substantiates the validation of Hypothesis 3.” (LINE 962-966)*

*“This finding suggests that preventing financial disintermediation positively impacts bank liquidity generation. In summary, digital transformation can mitigate financial disintermediation, thereby enhancing liquidity creation. This suggests that financial disintermediation serves a partial intermediary role in the process of improving the liquidity creation capacity associated with banks' digital transformation. Hypothesis 4 has been validated.” (LINE 1024-1029)*

Q3: Both the introduction and conclusion sections could be improved. The research problem needs to be articulated more clearly, and the practical implications of the study should be emphasized further. Please consider these suggestions in your revised version.

Response: We have revised the introduction section of the paper to highlight the main theme and significance of the study more prominently. We have also modified the conclusion section to provide a more in-depth description of the study's focus and significance. Specifically as follows：

*“As China's economy has entered a new normal, the increasing downward pressure on economic performance and the diminishing capacity of the banking system to support the real economy have become undeniable realities. The diverse financial needs of China's real economy remain inadequately addressed, with financing difficulties and elevated costs impeding high-quality economic development. According to modern financial intermediation theory, the liquidity creation function of commercial banks serves as a primary mechanism for supporting the real economy (Berger and Bouwman, 2009). This indicates that within China's financial system, which is predominantly characterized by commercial banks, the effectiveness of financial services in serving the real economy is primarily reflected in the level of bank liquidity creation. In light of the challenges posed by the novel coronavirus epidemic, China's real economy is grappling with difficulties in debt repayment, financing expansion, and capital turnover, necessitating that banks and other financial institutions provide sufficient liquidity. In response to these issues, regulatory authorities have implemented several policies in recent years, including allowing banks that meet inclusive finance assessment criteria to benefit from a preferential deposit reserve ratio and reducing excess deposit reserves to signal guidance for credit expansion. These measures have alleviated the short-term funding shortages in the real economy; however, this issue remains challenging to resolve substantively. Consequently, research on enhancing bank liquidity creation is not only a critical factor in addressing the financing dilemmas faced by the real economy in the short term but also an essential pathway for China's medium- and long-term financial and economic development.*

*It is noteworthy that, in recent years, the ongoing integration of digital technology with traditional financial services has enabled banks to systematically transform their product and service models through the application of digital technologies such as big data. As information technology increasingly converges with financial services, banks are also beginning to explore new development models within the realm of the digital economy. For instance, in 2023, the total fintech investment among China's six major banks reached 122.822 billion yuan, reflecting a growth of 5.38%. China Construction Bank leveraged digital technology to establish a comprehensive service platform for rural revitalization, providing nearly 100 billion yuan in agriculture-related credit to rural customers by the end of 2023. Meanwhile, China Merchants Bank has enhanced its digital risk management capabilities and efficiency through the development of an intelligent risk control engine, achieving a business intelligence warning coverage rate of 100% and streamlining digital processes that reduced the average mortgage loan processing time from one month to just 2.7 days. Thus, can the digital transformation of commercial banks mitigate the liquidity shortages faced by Chinese commercial banks? If such an effect exists, will it vary among different institutions? Clearly, these questions warrant further investigation.” (LINE 82-121)*

*“In the context of slowing economic growth exacerbated by the impact of the novel coronavirus pandemic, addressing the liquidity shortage faced by China's commercial banks has become a critical component in enhancing the transmission mechanism of monetary policy and ensuring that the financial system effectively supports the real economy. Building on this foundation, this paper first examines the specific mechanisms through which banks' digital transformation influences their liquidity creation levels. It empirically analyzes data from 127 commercial banks in China spanning the years 2010 to 2021, ultimately drawing the following key conclusions: First, an increase in the level of digital transformation among banks generally enhances their liquidity creation capacity, thereby facilitating a more effective support of the real economy by the financial system. This conclusion remains robust even after controlling for endogeneity and conducting various robustness tests. Second, the results of the transmission channel tests indicate that banks' digital transformation can optimize loan loss provisions, enhance their risk tolerance, and mitigate financial disintermediation. Consequently, banks are able to expand profit margins within the confines of risk management, thereby improving their liquidity creation levels. Third, from an external environmental perspective, the positive impact of banks' digital transformation on liquidity creation is more pronounced in regions with superior infrastructure development and a lesser influence from emerging digital financial formats. In regions with robust digital infrastructure, commercial banks can leverage favorable conditions to facilitate their digital transformation and enhance competitiveness, thereby promoting liquidity creation. In areas where the influence of emerging digital financial formats is limited, the digital transformation of commercial banks serves as a more effective alternative to traditional credit models, further fostering liquidity generation. From the perspective of internal conditions, the positive impact of digital transformation on liquidity creation is more pronounced in banks with substantial resource endowments, particularly large state-owned and joint-stock banks. This is attributable to their advanced technological capabilities in both hardware and software, as well as their ability to leverage superior channel resources and information security advantages. Consequently, these institutions are better positioned to capitalize on the scale benefits afforded by digital transformation, thereby enhancing their levels of liquidity creation.” (LINE 1487-1514)*

This letter is our point-by-point response to the comments raised by the reviews. The comments are produced and our responses are given directly afterward in a different color from the authors in the revised copy.

We would also like to thank you for allowing us to resubmit a revised copy of the manuscript.

We hope that the revised manuscript may address the concerns raised by the reviewers and will have the opportunity to be published in PLOS ONE.
