# Supplementary material for: Digital transformation and liquidity creation in commercial banks: Evidence from the Chinese banking industry
Source: PLoS One. 2025 Feb 13;20(2):e0318785. doi: 10.1371/journal.pone.0318785 (PMC11825035; doi:10.1371/journal.pone.0318785)
Supplement: Attachment — Submitted filename: Response to Reviewer4-1130.docx [file pone.0318785.s004.docx]

**Response to Reviewer 4 Comments**

Dear Editors and Reviewers:

Thank you very much for taking your time to review and comments concerning our manuscript entitled “Digital transformation and liquidity creation in commercial banks: evidence from the Chinese banking industry”. Those comments are all valuable and very helpful for revising and improving our paper. We have meticulously contemplated and analyzed the comments and suggestions provided by the reviewers and have revised the paper based on their feedback. Specifically, the following is a detailed account of the changes we have made in response to the reviewer comments (in the revised version, we have highlighted the changes in red font). The main changes in the paper and the responds to the reviewer #4 are as follows:

Q1: The topic is of significant interest, and the research problems are clearly articulated. Identifying research gaps is well-justified, and the literature review adequately supports the hypothesis formulation. However, a point of concern arises regarding the decision to exclude only the year 2020 to mitigate the impact of the COVID-19 pandemic on the robustness tests. Given that the effects of the pandemic persisted until 2022, the rationale for this exclusion warrants further clarification.

Response: We thank reviewer for reminding us this important point. Through empirical analysis and discussion, we believe that it is more appropriate to exclude the data of 2020 in this paper. For this, we offer the following explanation:

Firstly, 2020 was a crucial year for the outbreak of the COVID-19 pandemic, and it was also the year when society was most severely impacted. Due to lockdowns and isolation measures, China's supply chains were disrupted, consumer demand plummeted, and bank liquidity, asset quality, and credit demand experienced significant fluctuations. For example, China's GDP increased from 98.65 trillion yuan in 2019 to 101.36 trillion yuan in 2020, with economic growth slowing from 6% to 2.2%. Therefore, 2020 should be prioritized for exclusion.

Secondly, in 2021, the global economy continued to be affected by the fluctuations of the pandemic, but the economy and financial markets began to recover. For example, China's GDP in 2020 was 101.36 trillion yuan, and economic growth was severely stifled, with a mere 2.2% growth rate. In 2021, China's economic recovery trend was obvious, with GDP reaching 114.92 trillion yuan and an 8.4% growth rate. In 2022, the global economy also began to recover, and China gradually loosened economic and trade restrictions. At the same time, digital economy grew rapidly. The liquidity and credit demand of Chinese commercial banks began to approach normal levels. In 2022, the total amount of yuan loans increased by 21.31 trillion yuan, an increase of 136 billion yuan compared to the previous year. The impact of digital transformation on the economy may have become apparent in 2021-2022. Therefore, we suggest retaining the data of 2021 and excluding the data of 2020.

To verify the above reasons, this paper re-examined the impact of excluding 2020-2021 data, as shown in Table 1 (column 2). The results show that the Tdi coefficient is significantly positive at the 5% level, and the conclusion that bank digital transformation promotes liquidity creation remains unchanged, and the results are still robust. Due to space limitations, this part of the test is not presented in the main text.

Table 1 Changes to Sample Period

| Variable | Exclude the data from 2020 | Exclude the data from 2020-2021 |
| --- | --- | --- |
|  | The early stages of the COVID-19 pandemic | the early stages of the COVID-19 pandemic and the transition period |
|  | （1） | （2） |
| *Tdi* | 0.037^*^  (1.91) | 0.041^**^  (2.52) |
| *Ind* | 0.066^*^  (1.77) | 0.072^*^  (1.65) |
| *M2* | 0.061^*^  (1.82) | 0.057^**^  (2.02) |
| *Gdpg* | 0.084^***^  (3.77) | 0.079^***^  (2.79) |
| *Npl* | -0.185  (-0.35) | -0.173  (-0.37) |
| *Tcr* | -0.088^*^  (-1.78) | -0.093^*^  (-1.65) |
| *Size* | 0.112^***^  (3.11) | 0.106^***^  (3.26) |
| *Nir* | 0.079^*^  (1.74) | 0.0.102^*^  (1.75) |
| *Roe* | 0.055  (1.46) | 0.052^*^  (1.69) |
| Individual Effect | Yes | Yes |
| Time effect | Yes | Yes |
| Regional effect | Yes | Yes |
| *N* | 827 | 780 |
| *R^2^* Value | 0.313 | 0.295 |

Note: ***, **, * indicate statistical significance at the 1%, 5%, and 10% levels (two-tailed test); parentheses represent t-values; we use robust standard error clustered by bank level for heteroscedasticity-adjusted clustering.

This letter is our point-by-point response to the comments raised by the reviews. The comments are produced and our responses are given directly afterward in a different color from the authors in the revised copy.

We would also like to thank you for allowing us to resubmit a revised copy of the manuscript.

We hope that the revised manuscript may address the concerns raised by the reviewers and will have the opportunity to be published in PLOS ONE.
